# What’s childhood asthma in French Guiana? A cohort study based on children referred for allergology consultations at the Cayenne hospital center

**DOI:** 10.3389/fpubh.2023.1198937

**Published:** 2023-09-07

**Authors:** Chiméne Maniassom, Antoine Defo, Frédéric De Blay, Narcisse Elenga

**Affiliations:** ^1^Department de Pediatrie, Hôpital de Cayenne “Andrée Rosemon”, Cayenne, French Guiana; ^2^Pôle de Pathologie Thoracique Hôpitaux Universitaires de Strasbourg, Place de l’Hôpital, Strasbourg, France

**Keywords:** asthma, allergy, *Blomia tropicalis*, children, French Guiana

## Abstract

**Background:**

Asthma is a multifactorial chronic disease, whose most frequent etiology is allergy, especially to *Blomia tropicalis*. In French Guiana, the childhood prevalence of Blomia T sensitization is unkwown. The aim of this study was to determine the proportion of sensitization to Blomia T and other mites in asthmatic children, and to describe the characteristics of childhood asthma in French Guiana.

**Methods:**

A retrospective cohort study focused on children from 0 to 18 years of age, followed for asthma at the Department of Pediatrics of the Cayenne Hospital Center in French Guiana. All asthmatic children followed by the same paediatric allergist were systematically skin-tested with Bt total extract, and Bt-specific IgE tests were additionally performed to confirm specific sensitization. All follow-up variables were collected from medical records. The outcome was sensitization to *Blomia tropicalis* and other allergens, and the explanatory variables were those of asthma follow-up. Patients were categorized into *Blomia tropicalis* sensitization yes/no. Logistic regression analysis was used to assess the relationship between follow-up variables and the outcome.

**Results:**

302 patients were followed: 177 cases of allergic rhinitis, 135 allergic conjunctivitis, 105 atopic dermatitis, 153 food allergy, and 14 cases of drug allergy. Poly-allergy (respiratory, food, skin, and medicinal) was present in 239 children. There were 158 children followed for asthma, of whom 103 (65%) were sensitized to *Blomia tropicalis*. The median age of the asthmatic children sensitized to *Blomia tropicalis* was 7 years, and 3 years for those who were not sensitized (*p* < 0.001). Among the girls (*n* = 58), 67% were sensitized to *Blomia*; 97 (92%) asthmatic children co-sensitized to *Blomia tropicalis*, *Dermatophagoides pteronyssinus,* and *Dermatophagoides farinae*. Multivariate analysis showed that the childhood asthma in French Guiana is characterized by a median age of 7 years (*p* < 0.001), a high prevalence of *Blomia tropicalis* (*p* < 0.001), co-sensitization to other mites (p < 0.001), and a high prevalence of co-sensitization to cockroaches (*p* = 0.006). The area under the ROC curve was close to 0.9, confirming the quality of our model.

**Conclusion:**

In French Guiana, asthma is characterized by a high prevalence of Blomia tropicalis sensitization.

## Highlights

In this study, we characterized the pattern of childhood asthma in a tropical setting. We demonstrated that the childhood asthma is characterized by a median age of 7 years, a high prevalence of Blomia tropicalis sensitization, a co-sensitization to other mites, and a high prevalence of co-sensitization to cockroach allergens.

## Introduction

House dust mites are part of the arthropods phylum and the arachnids class (1). Their size varies from 170 to 500 μm and their lifetime is 3 months on average. Their passage from egg to larva and then to protonymph and tritonymph takes 1 month. Their feces weigh 10–40 μg and 20 feces are eliminated per day (1). They grow in hot and humid environments for which the main reservoir is the domestic environment. The main mites are *Dermatophagoides pteronyssinus* (DP), *Dermatophagoides farinae* (DF), *Blomia tropicalis* (BT), and *Tyrophagus putrescentiae* (Tyr p 4).

*Blomia tropicalis* is a mite species in the superfamily Glycyphagidae. Originally described as a storage mite, it is now considered a tropical and subtropical house dust mite.

Sensitization to this mite is very common in South America (2, 3) and in Southeast Asia (4, 5). Epidemiological studies have also found sensitization to this mite in Africa and Central America (6, 7).

Blo t 5 and Blo t 21 are the main allergens of *Blomia tropicalis* (8). Of the 25 IgE-binding proteins identified in *B. tropicalis*, 13 have been officially recognized and named internationally (9). Co-sensitization to other mites such as *Dermatophagoides pteronyssinus* and *Dermatophagoides farinae* is very common (8).

The involvement of *Blomia tropicalis* in allergic rhinitis and asthma is well described. It is also implicated in other allergic diseases (8).

In tropical Africa, sensitization to dust mites in children followed for respiratory allergy is common. B. T was present in 4–8% of patients, and it was associated with *Dermatophagoides pteronyssinus* and *Dermatophagoides farinae* in 86% of cases (9).

In French Guiana, a tropical territory characterized by poverty and poor social conditions, is the prevalence of Blomia T sensitization awareness as high as in other tropics?

The aim of this study was to determine the proportion of sensitization to *Blomia T*, *DF*, *DP*, and Tyr p 4 in asthmatic children as well to describe the characteristics of childhood asthma in French Guiana.

## Materials and methods

### Study setting

The entire territory of French Guiana covers 86,500 km^2^. The population was estimated to be 294,071 inhabitants on January 1, 2021. The Amazon rainforest, which includes several thousand plant species, covers a large part of French Guiana (96%). The population is, therefore, mainly concentrated on the coast in the Cayenne (61,268 inhabitants), Saint-Laurent-du-Maroni (42,612 inhabitants), and Kourou (25,685 inhabitants) conurbations (10). Guiana has an equatorial climate, with the temperature varying little during the year between 20 and 32°C. The humidity is very high, varying from 70 to 90%. Precipitation is abundant. French Guiana has four seasons: the main rainy season from April to June; the dry season from July to mid-November; a rainy season from late November to late February; and the short summer of March (11). This French territory in America is also characterized by the regular presence of dust from the Sahara. French Guiana, because it is part of France, combines the living conditions of both rich and poor countries. In French Guiana, there is only one pediatric allergist, and the pediatric department of the Cayenne hospital Center is the reference pediatric department for the entire territory of French Guiana. All children with allergies are then referred to this center.

### Type of study

We conducted a retrospective cohort study in children aged 0 to 18 years from all over French Guiana, followed from 05 July 2017 to 05 July 2020 at the Department of Pediatrics of the Cayenne Hospital Center for allergic disease.

### Routinely collected data

All children were skin prick tested with Bt-total extract and Bt-specific IgE testing was performed in addition to confirm specific sensitization.

The parameters collected, established during the consultation were:

- Age, sex, growth, vaccination status.
- We also collected certain variables such as “abnormal growth” and “psychomotor retardation” as medical histories (reported in the files), although an association with asthma status or BT sensitization may not be apparent.
- Associated pathologies and co-allergy (respiratory, food, skin, and drug).
- Evaluation of asthma before the start of specific management: control, possible ongoing background treatment, symptoms of nocturnal or daytime asthma, exercise-induced asthma (EIA), number of annual attacks and hospitalization, and asthma severity.
- Assessment for skin and blood sensitization to the main childhood respiratory allergens: mites (*Blomia tropicalis*, *Dermatophagoides pteronyssinus*, and *Dermatophagoides farinae*, *Lepidoglyphus destructor*), cats, dogs, cockroaches, *Aspergillus fumigatus*, *Alternaria alternata*, and horses.
- Chest X-ray, flow-volume loop, anemia assessment.
- Treatment initiated: antiacarian pillow cover, inhaled corticosteroids, combination of long-acting corticosteroid-bronchodilator (BDLA), antileukotrienes, anti-allergenic immunotherapy (ATI), biotherapy, and therapeutic education
- Evaluation after 1 year of follow-up with: the number of annual attacks and hospitalizations, asthma control, and flow-volume loop.

### Evaluation criteria

The primary endpoint was the number of children with asthma sensitized to *Blomia tropicalis*.

The secondary outcomes were:

- The number of asthmatic children co-sensitized to *Blomia tropicalis* and other mites.
- The number of children with asthma co-sensitized to other allergens (shrimp, mildew, cockroaches).
- Asthma severity was classified as remission, intermittent, mild persistent, moderate persistent and severe persistent.
- Level of asthma control after 1 year of management. Control was classified into 3 levels: unacceptable, acceptable and optimal. The parameters defining acceptable control are adapted from the French national protocols.
- The number of children with asthma with a favorable outcome.
- The number of children lost to follow-up.
- Final change was categorized as normal progression, poor compliance and lost to follow-up.

### Sample size calculation

The number of subjects required to detect a 20% relative difference in sensitisation to Blomia tropicalis, with a power of 80% and a false positive rate of 5%, was 148, or 74 in each group.

### Statistical analysis

These data were analyzed using STATA software (Stata Statistical Software: Release 15. College Station, TX: StataCorp LP).

The results of the descriptive analysis are expressed as percentages and numbers for qualitative variables. Percentages were calculated by dividing the number of subjects by the total number of subjects in each group. Quantitative variables are described as a median (25th–75th percentile) for non-Gaussian variables or as an average (± 2DS) for variables with a normal distribution. Categorical variables were compared using the *χ*^2^ test or Fisher exact test (as appropriate) and continuous variables were compared with the Mann–Whitney ∗U∗ test. *p* values < 0.05 were considered statistically significant. The alpha risk was set at 5%.

The Kolmogorov Smirnov test was applied to the age distribution in the B.T. sensitized/non-sensitized groups.

The outcome was sensitization to BT and other allergens and the explanatory variables were those of asthma follow-up.

In a first step, we used each possible clinical measurement and determinant of the outcome sensitization to B.T. yes/no in a univariate logistic model. Then, in a second step, a subsequent multivariate logistic analysis was performed with a selection of characteristics that were statistically significant in the univariate analyses.

We defined sensitivity (Se) as the proportion of true positives among those to be screened and specificity (Sp) as the proportion of true negatives among non-patients. The positive predictive value or PPV represents the probability of being affected by a disease in the event of a positive test. The negative predictive value is the probability of not being affected by a disease when the test is negative. A receiver operating characteristic (ROC) curve was generated to test our multivariate model.

### Regulatory authorizations

In terms of regulatory aspects, using the consultation files (computerized and paper medical files) we created an anonymized database in an Excel spreadsheet. This database has been declared to the CNIL (Commission Nationale Informatique et Libertés) and we have obtained approval from the Ethics Committee of the Cayenne Hospital (N°013–2022).

## Results

**Figure 1:**
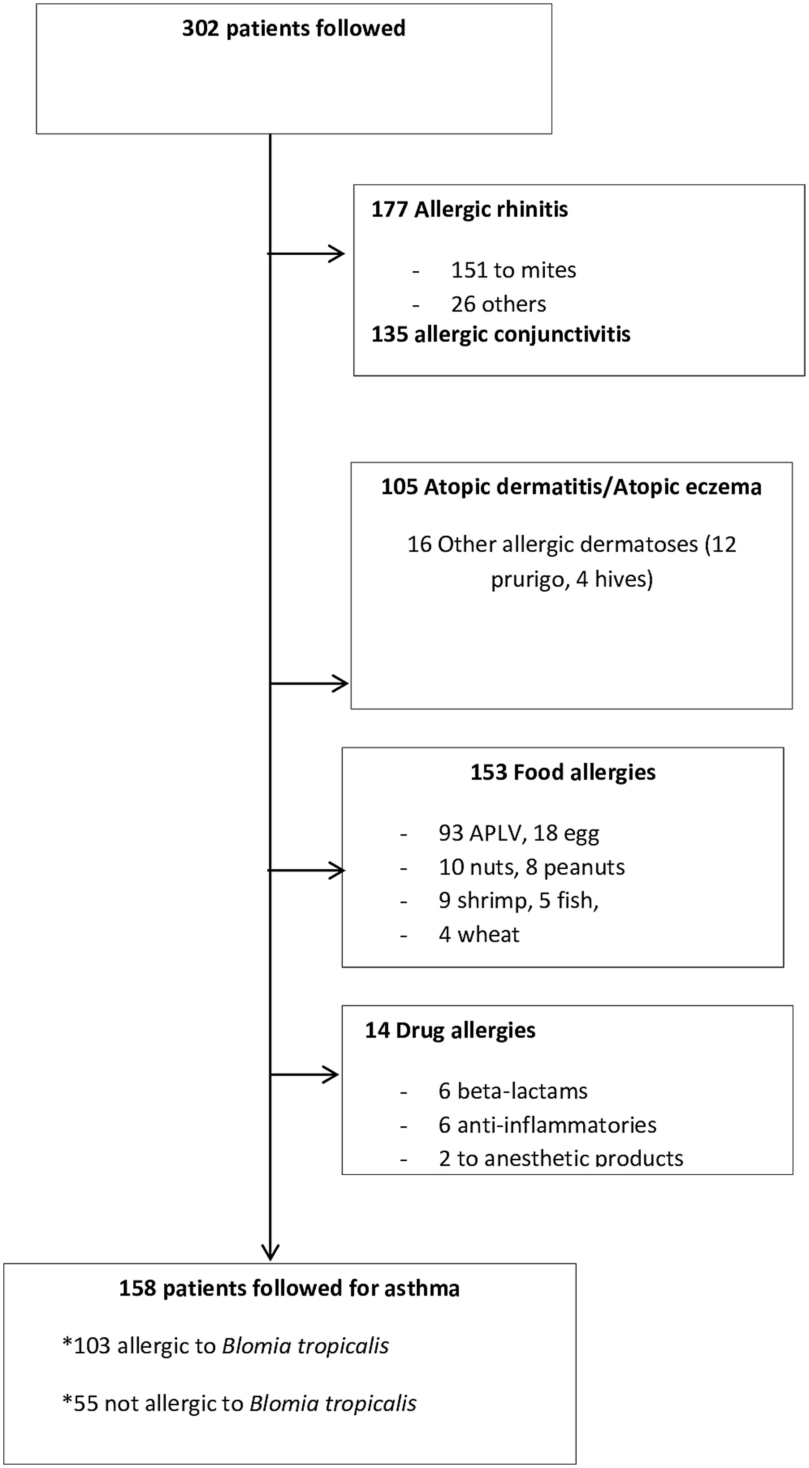
Characteristics of the 302 patients monitored.

From July 05, 2017 to July 05, 2020, 302 patients were referred to us for pathologies related to allergies. Among these, there were 177 cases of allergic rhinitis, 135 cases of allergic conjunctivitis, 105 cases of atopic dermatitis, 153 cases of food allergy, and 14 cases of drug allergy (Figure 1). Poly-allergy (respiratory, food, skin, and medicinal) was present in 239 children. There were 158 children who were followed for asthma, of whom 103 (65%) were sensitized to *Blomia tropicalis*.

Table 1 lists the characteristics of the children being followed for asthma. The median age was 7 years for children sensitized to *Blomia tropicalis*, and 3 years for children not sensitized to *Blomia tropicalis* (*p* < 0.001). There were 100 boys (63%) and 58 girls (37%). Among the girls, 67% were sensitized to *Blomia*.

**Table 1:** Clinical and biological characteristics of the asthmatic patients.

Variables	*Blomia* + *N1* = 103	*Blomia- N2* = 55	*p*
Age (Years, median, IQR)*	7 [5–11]	3 [2–5]	<0.001
Sex F (*n*, %)	37 (36)	21 (38)	0.8
Medical history prior to follow-up			
Abnormal growth (*n*, %)	22 (21)	8 (15)	0.3
Psychomotor delay (*n*, %)	4 (4)	1 (2)	0.5
Associated pathology (*n*, %)	101 (98)	43 (78)	0.001
Asthma control at baseline (*n*, %)	19 (18)	9 (16)	0.7
Ongoing of background treatment at the beginning of follow-up	33 (32)	15 (27)	0.5
Nocturnal cough (*n*, %)	68 (66)	32 (58)	0.3
Morning cough (*n*, %)	33 (32)	16 (29)	0.7
Exertion-induced asthma (*n*, %)	59 (57)	24 (44)	0.2
Diurnal symptoms (*n*, %)	10 (10)	3 (5)	0.4
Annual number of exacerbations before follow-up (median, IQR)	4 [3–7]	4 [3–6]	0.9
Annual number of hospitalizations before follow-up (median, IQR)	0 [0–1]	0 [0–1]	0.9
Follow-up outcomes			
Asthma control/follow-up (*n*, %)			0.09
Controlled asthma	52 (50)	24 (44)	
Partially controlled asthma	39 (38)	19 (35)	
Uncontrolled asthma	6 (6)	4 (7)	
Lost to follow-up	6 (6)	8 (15)	
Final change (*n*, %)			0.4
Normal progression	83 (81)	43 (78)	
Poor compliance	10 (10)	4 (7)	
Lost to follow-up	10 (10)	8 (15)	
Asthma severity (*n*, %)			0.8
In remission	1 (1)	3 (5)	
Intermittent	19 (18)	6 (11)	
Mild persistent	50 (49)	27 (49)	
Moderate persistent	33 (32)	18 (33)	
Severe persistent	0 (0)	1 (2)	
Annual number of crises after 1 year of follow-up (median, IQR)	0 [0–1]	1 [0–1]	0.2
Main identified allergens			
D. pteronyssinus (*n*, %)	97 (94)	8 (15)	<0.001
D. farinae (*n*, %)	97 (94)	6 (11)	<0.001
Cockroaches (*n*, %)	57 (55)	8 (15)	0.01
*Aspergillus fumigatus* (*n*, %)	14 (14)	0	0.9
Alternaria alternate (*n*, %)	15 (15)	0	0.8

IQR, interquartile range.

*The structural age groups used for analysis are infants (0–3 years), children (4–12 years) and adolescents (>12 years).

There were 97 asthmatic children (92%) who were co-sensitized to *Blomia* and DP,

And 97 children (94%) were also co-sensitized to *Blomia* and DF.

Among the asthmatic children co-sensitized with *Blomia* and other mites:

- 57 (88%) were to cockroaches.
- 14 to *Aspergillus fumigatus*.
- 15 to *Alternaria alternata*.

Of the asthmatic children co-sensitized to *Blomia* and food allergens, 7 were sensitized to shrimp and 5 to peanuts, with 3 cases in the control group. Anemia was noted in 4 cases in the *Blomia* group and in 5 cases in the control group. We did not observe any sensitization in Tyr p 4.

After 1 year of follow-up, there were 52 cases (68%) of controlled asthma in the patients sensitized to *Blomia* compared to 24 patients (32%) without sensitization. This difference was, however, not statistically significant (*p* = 0.09).

Of the asthmatic children followed, 83 (66%) were in the *Blomia*-sensitized group compared to 43 (34%) in the second group. This difference was not statistically significant (*p* = 0.4).

At the end of 1 year of follow-up, 10 children (56%) in the *Blomia*-sensitized group and 8 (44%) in the non-sensitized group were lost to follow-up. This difference was, however, not statistically significant (*p* = 0.4).

In addition, all patients had a normal chest X-ray. The proportion of children with an obstructive ventilatory disorder decreased from 24% before to 14% 1 year after starting the treatment. It should be noted that 60% of children did not succeed with spirometry. A prescribed mite cover has been purchased by 56% of patients.

At the therapeutic level, inhaled corticosteroids were used in 56% of cases in the *Blomia* group versus 44% in the control group; corticosteroid therapy plus a long-acting bronchodilator was used in 77% of cases in the *Blomia* group versus 23% in the second group; antileukotrienes were used in 31 (66%) of cases in the *Blomia* group versus 16 (34%) in the second group.

Antiallergenic immunotherapy for the three allergens (DP + DF + BT) was used in 27 cases (96%) in the *Blomia* group compared to 1 (4%) in the second group.

Biotherapy was used in a single patient sensitized by *Blomia*. The annual number of exacerbation episodes decreased from 4 [3–7] to 0 [0–1] cases in the *Blomia* group compared to 4 [3–6] to 1 [0–1] in the second group.

The multivariate analysis comparing the two groups of asthmatic children (Table 2) showed that childhood asthma in French Guiana is characterized by:

- A median age of 7 years.
- A high prevalence of *Blomia tropicalis*.
- High prevalence of BT co-sensitization to acarians: DP and DF.
- High prevalence of co-sensitization to cockroaches.

**Table 2:** Multivariate analysis comparing the two groups of children with asthma.

Variables	*Blomia*+	*Blomia*-	AOR	*p*
Age (median, IQR)	7 [5–11]	3 [2–5]	5.0 [2.3–10.9]	<0.001
Associated pathology (*n*, %)	101 (98)	43 (78)	10.6 [1.1–109.1]	0.04
*Dermotophagoides pteronyssinus*	97 (94)	6 (11)	49.9 [12.2–203.4]	<0.001
(n, %)				
Cockroaches (*n*, %)	57 (55)	8 (15)	0.2 [0.1–0.7]	0.006
Asthma control/follow-up (*n*, %)	52 (50)	24 (44)	3.1 [0.9–10.2]	0.07

**Sensitivity**	**Specificity**	**PPV**	**NPV**
93%	73%	86%	85%

AOR, adjusted odds ratio.

IQR, interquartile range.

PPV, positive predictive value.

NPV, negative predictive value.

The multivariate logistic analysis was performed with a selection of characteristics that were statistically significant in the univariate analyses. The adjustment variable here is gender.

**Figure 2:**
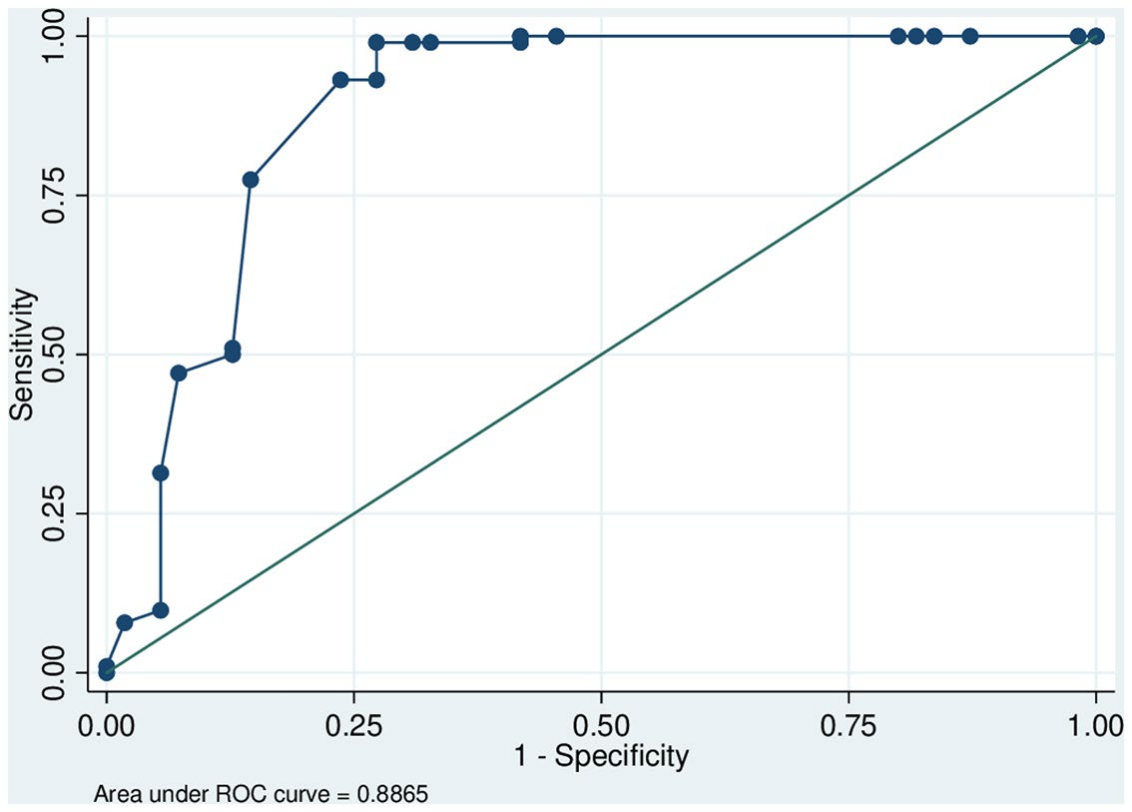
ROC (receiver operating characteristic) curve to test our multivariate model. The area under the ROC curve was close to 1, confirming the quality of the model. ROC analysis is used here to quantify the accuracy with which our medical diagnostic test (child with median age of 7 years, *Blomia tropicalis*, co-sensitization to *Dermatophagoides pteronyssinus*, and cockroaches) distinguishes children with allergic asthma in French Guiana.

Our model exhibited a sensitivity of 94% and a specificity of 73%. The PPV was 86% and the NPV 85%. The area under the ROC curve was close to 0.9, confirming the quality of our model (Figure 2).

## Discussion

Our study showed that childhood asthma in French Guiana is characterized by a median age of 7 years, a high prevalence of sensitization to Blomia tropicalis, co-sensitization to other house dust mites and a high prevalence of co-sensitization to cockroach allergens. The median age was 7 years for children sensitized to Blomia tropicalis, and 3 years for children not sensitized to Blomia tropicalis. This observation can be explained in two ways. One possibility is that Blomia tropicalis sensitization is a late event, and that the children not sensitized to Blomia tropicalis are simply studied at an early phase of their clinical history, when this specific sensitization has not yet occurred. Alternatively, this finding may indicate that two populations of asthmatic children exist, differing on their risk of sensitization to Blomia tropicalis. Since those that are sensitized to BT have frequently co-sensitization to other agents, the first possibility seems to fit the data better.

As found in our study, co-sensitization to the allergens of other mites such as DP and DF is very common (12). The origin of this co-sensitization remains to be determined, although it is possible that the mechanism is multifactorial, with a common environmental exposure and potentiation of allergy by the parasite *Ascaris lumbricoides* (13, 14). It is likely that the parasite *Ascaris lumbricoides* plays a role in sensitization and clinical expression to *B. tropicalis*. Thus, in the serum of asthmatic patients, *B. tropicalis* and Der p inhibit 83.3 and 79.0% of Ascaris-related IgE, respectively. The tropomyosin of these mites inhibits 85*%* of IgE bound to roundworm. IgE directed against rBlo t 10 identified a 40 kDa allergen on *Ascaris*, which turned out to be tropomyosin (15). In addition, infestation by *A. lumbricoides* may act by potentiating the Th2 response to the allergen and thus increase allergenicity to mites including *B. tropicalis* (14). Among the asthmatic children co-sensitized with Blomia and other mites: 57 (88%) were sensitized to cockroach allergens. Furthermore, since such sensitization might be suggestive of exposure to these allergens in the home, due to poor hygiene standards, it is worth mentioning that other studies, carried out in New York City poor neighborhoods have shown a similar pattern, which was accompanied by sensitization to rat allergen or by the observation of an association between infection with Ascaris lumbricoides. In the present study the possibility of allergy to rat allergens could not be identified, although it might be relevant to the interpretation of the data. This possibility is reinforced by the observation of an association between infection with Ascaris lumbricoides, a major marker for poor hygiene, and sensitization to BT.

In accordance with the data in the literature, our study population was male-dominated. Indeed, the predominance during childhood is male and then evolves into a female predominance after puberty (16).

The severe childhood asthma phenotype, associated with allergic comorbidities such as eczema, allergic rhinitis, and food allergies, is more frequently encountered in boys, according to Just et al. (17, 18). Of the asthma patients included, 65% were sensitized to dust mites. French Guiana is an area well known for its humidity, which favors the emergence of *Blomia tropicalis.* These results are in line with what has been reported in countries with limited resources, especially in regions where there are optimal conditions for their proliferation (6, 19, 20, 22, 23). Indeed, between 65 and 130 million people worldwide are sensitized to dust mites, and this sensitization affects 50% of asthmatics (24). Out of all allergens, dust mites are known to be a major risk factor for developing asthma and rhinitis (25). We found that asthma severity did not differ according to sensitization to *B. tropicalis*. On the other hand, To My et al. (26) noted more severe asthma in patients who had positive skin tests than in those who had negative tests, with a very significant difference (*p* < 0.0005). Asthma control also did not differ according to sensitization to *B. tropicalis*.

Since exposure to dust mites has a major impact on the occurrence of asthma, it is important to offer patients avoidance measures. Some essential measures must be followed to reduce the allergenic load of dust mites, such as ventilation and storage in the room; removal of carpets; cleaning the floor once or twice a week; not using traditional brooms, which make dust fly, but instead using a terry broom or a damp cloth or a vacuum cleaner with specific filters (HEPA); washing sheets every week, if possible at 60°; replacing kapok pillows with synthetic pillows, and washing these pillows regularly; and exposing synthetic mattresses to the sun or careful vacuuming or wrapping with a specific cover to avoid contact with dust mites. Chemical acaricides are also used here where there is a high prevalence of children infected with helminthiasis (27). The management of childhood asthma is well codified, and we have taken into account the recommendations of the High Authority of Health and the Francophone Pneumology Society (28) as well as those of the Global Initiative for Asthma (GINA) (29, 30).

Our study has some limitations. First of all, the number of patients studied is small. But this collection is exhaustive because it concerns all patients who have been referred to specialized pediatric allergology consultations. As fecal examination was not routinely performed, we could not confirm the role of *Lumbricoides ascaris* in sensitization to *B. tropicalis*. Also, some patients were lost to follow-up, which did not allow all their data to be exploited. In addition, we could not study the effect of other environmental factors such as climate, air quality, rainfall, and viral epidemics on the occurrence of asthma attacks. Despite these limitations, this is the first study in Guiana to evaluate the prevalence of *Blomia tropicalis* in children and by the same doctor. This study allowed characterization of asthmatic children in French Guiana, using ROC curves, from the results of the multivariate analysis.

## Conclusion

In this study, we characterized the pattern of childhood asthma in a tropical setting. We demonstrated that the childhood asthma is characterized by a median age of 7 years, a high prevalence of Blomia tropicalis sensitization, a co-sensitization to other mites, and a high prevalence of co-sensitization to cockroach allergens. These elements should be investigated in the initial assessment of asthmatic children in order to improve the diagnostic quality and the therapeutic management.

## Data availability statement

The raw data supporting the conclusions of this article will be made available by the authors, without undue reservation.

## Ethics statement

The studies involving humans were approved by the Ethics Committee of the Cayenne Hospital (N°013–2022). Rue des Flamboyants BP 6006–97306 Cayenne Cedex. The studies were conducted in accordance with the local legislation and institutional requirements. Written informed consent for participation was not required from the participants or the participants’ legal guardians/next of kin in accordance with the national legislation and institutional requirements.

## Author contributions

CM collected the data and wrote the first draft of this article. AD and FB critically reviewed it for important intellectual content. NE analyzed the data and approved the final version for publication. All authors contributed to the article and approved the submitted version.

## Conflict of interest

The authors declare that the research was conducted in the absence of any commercial or financial relationships that could be construed as a potential conflict of interest.

## Publisher’s note

All claims expressed in this article are solely those of the authors and do not necessarily represent those of their affiliated organizations, or those of the publisher, the editors and the reviewers. Any product that may be evaluated in this article, or claim that may be made by its manufacturer, is not guaranteed or endorsed by the publisher.
